# *Bru*SIC: a novel selective medium for the primary isolation of *Brucella* in veterinary samples

**DOI:** 10.1128/spectrum.01759-22

**Published:** 2022-11-03

**Authors:** Sara Mena-Bueno, Irati Poveda-Urkixo, Daniel Asensio, Iñaki Echarte, Ana Zabalza-Baranguá, María Jesús Grilló

**Affiliations:** a Instituto de Agrobiotecnología (CSIC-Gobierno de Navarra), Mutilva Baja, Spain; b Universidad Pública de Navarra, Mutilva Baja, Spain; c Reactivos para Diagnóstico, S.L. Josep Tura, Sentmenat, Spain; UJF Grenoble and CHU Grenoble

**Keywords:** *Brucella*, primary isolation, selective culture media, veterinary samples, activated charcoal, calf serum

## Abstract

Brucellosis, a re-emerging zoonotic infection, threatens animal welfare and public health with serious economic consequences. A definitive diagnosis requires Brucella isolation by culturing field specimens in specific media. This study aimed to (i) assess the effectivity of recommended Farrell’s médium (FM) and CITA medium (CM) for the isolation of four Brucella melitensis strains (16M, Rev1, and the 16MΔ*wzm* and Rev1Δ*wzm* in-frame deletion mutants) with variable susceptibility to polymyxins; (ii) develop a Brucella selective medium (BSM) suitable for these strains; (iii) test BSM, FM, and CM with other Brucella species; and (iv) develop an improved selective culture medium (*Bru*SIC) for all brucellae, including B. abortus bv1. The four B. melitensis strains were strongly inhibited in FM and (except Rev1) CM. Since Rev1Δ*wzm*’s CM inhibition was due to a synergistic effect of colistin and vancomycin, we formulated BSM with half the concentrations of both antibiotics, achieving a similar growth of B. melitensis to blood agar base (BAB) and an inhibition of contaminant microorganisms comparable to CM; CM performance was surpassed by BSM for the primary isolation of B. melitensis when tested in 1,789 real sheep samples. For other brucellae, BSM and CM were more inhibitory than FM for B. abortus bv1 when using plates immediately after preparation but not after ≥4 weeks of storage. To address this, we developed the improved solid medium *Bru*SIC by replacing the calf serum in BSM with activated charcoal. *Bru*SIC yielded faster colony growth than BSM and CM and similar CFU numbers than BAB (including for B. ovis in BAB-Serum) and inhibited accompanying microorganisms in sheep and cow samples as effectively as BSM.

**IMPORTANCE** Farrell’s medium (FM) and CITA medium (CM), recommended for Brucella isolation in animal samples, are inhibitory for certain strains. A reformulated Brucella selective medium (BSM), containing half the CM vancomycin and colistin concentrations, improved the isolation of B. melitensis, but not Brucella abortus bv1. A novel Brucella selective culture medium (*Bru*SIC), in which calf serum is replaced by activated charcoal, retains the selectivity and improves the productivity of BSM and CM. *Bru*SIC allows the growth of all brucellae faster than in CM or BSM, and at CFU number equivalent to BAB supplemented by calf serum, including B. abortus bv1 and the serum-dependent Brucella ovis. Due to its performance and reduced cost, *Bru*SIC represents an added-value alternative to the existing selective culture media for these bacteria.

## INTRODUCTION

Brucellosis is a worldwide zoonosis whose etiological agents are bacteria of the genus Brucella, the most relevant species being B. melitensis, B. abortus, B. ovis, B. suis and B. canis. In livestock, the disease is characterized by abortion, sterility, and other reproductive disorders and is transmitted to animals and humans by bacterial shedding in placental and vaginal discharges, milk, and semen. The isolation of the pathogen from field samples is the gold standard test to confirm Brucella infection, but the primary isolation of slow-growing Brucella colonies is frequently hindered by fast-growing microorganisms from the environment and animal microbiota (1). Thus, selective culture media are a necessary tool to inhibit contaminant microorganisms and allow the isolation of Brucella from veterinary samples.

Among the available selective solid media (2–14), Farrell’s medium (FM) is widely recommended and applied, since it strongly inhibits fast-growing bacteria and its translucence facilitates the identification of Brucella morphology. However, FM contains nalidixic acid and bacitracin, which severely inhibit some Brucella strains (15–18), a drawback that has led to the formulation of modified FM (16) and CITA medium (CM) (17). Therefore, duplicate culturing in both FM and CM plates is currently the recommended method for the primary isolation of Brucella (1, 19). Nevertheless, susceptibility to polymyxins B (in FM) or E (colistin in CM) has been widely associated with attenuated Brucella mutants with exposed anionic phosphate groups of the lipid A lipopolysaccharide (LPS), such as rough LPS (R-LPS) mutants (20–28). In our laboratory, PCR overlapping was used to obtain two R-LPS vaccine candidates (Rev1Δ*wzm* and 16MΔ*wzm*) with high susceptibility to polymyxins (27) to be investigated for safety and efficacy in the natural host.

Before starting studies in sheep, we first tested the performance of FM and CM for the isolation of the polymyxin-susceptible mutants B. melitensis 16MΔ*wzm* and Rev1Δ*wzm*, as well as the respective parental 16M and Rev1. The high level of inhibition observed prompted us to formulate a Brucella selective medium (BSM), which was tested in 1,789 veterinary samples. The new solid medium inhibited contaminant microorganisms as effectively as CM and surpassed CM performance in the detection of both attenuated B. melitensis mutants. Unexpectedly, we also observed that BSM and CM were highly inhibitory against B. abortus bv1 strains, although only when using recently prepared plates and not after more than a month of storage. To overcome this inhibition, we further modified the BSM and developed a definitive Brucella selective improved culture (*Bru*SIC) medium, which increased the detection level of all the Brucella strains with restricted growth in FM, CM, and/or BSM. The new *Bru*SIC medium matched the other selective media in inhibiting accompanying contaminant microorganisms. Due to its performance and reduced cost, *Bru*SIC represents an added-value alternative to the existing selective culture media for these bacteria.

## RESULTS

### Growth of different *B. melitensis* strains in the recommended standard selective culture media.

**FIG 1:**
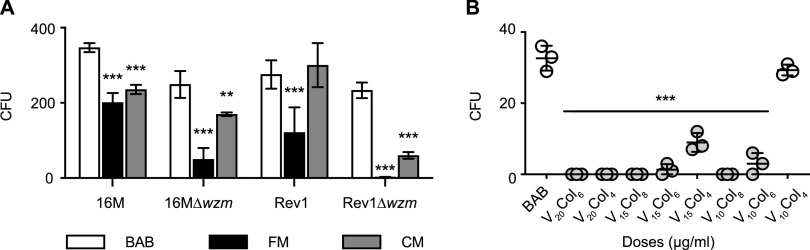
Growth of B. melitensis strains in selective Farrell’s medium (FM) and CITA culture medium (CM). (A) Number of CFU counted in Blood Agar Base No. 2 (BAB; control), FM, and CM by triplicate seeding 100 μL of a suspension containing ≈300 CFU of the corresponding Brucella strain, and plates incubation at 37°C for 14 days. The results are presented as mean numbers of CFU ± the SD (*n* = 3). (B) Number of CFU of Rev1Δ*wzm* in BAB and CM modified with different concentrations of vancomycin (V) and colistin (Col) (means ± the SD; *n* = 3). A Fisher least significant difference (LSD) test was performed (***, *P* ≤ 0.001; **, *P* ≤ 0.01) versus the BAB control.

** (i) CM improves the growth of *B. melitensis* with respect to FM but inhibits the growth of 16M, 16MΔ*wzm*, and Rev1Δ*wzm***. First, we assessed the growth of 16M, Rev1, and their in-frame deletion mutants, 16MΔ*wzm* and Rev1Δ*wzm*, susceptible to polymyxins (Table 1) in FM and CM versus Blood Agar Base No. 2 (BAB). As expected, the four B. melitensis strains tested, in particular the two rough mutants, were highly inhibited in FM versus BAB (*P* ≤ 0.001) (Fig. 1A). Also, the number of CFU detected was significantly higher in CM than in FM, but only Rev1 grew in CM at the levels of BAB.

**TABLE 1:** Brucella strains used in this study^*a*^

Strain	Characteristics	Origin
H38	B. melitensis bv1 virulent strain; S-LPS	IdAB-CSIC
16M	B. melitensis bv1 reference virulent strain; S-LPS	IdAB-CSIC
16MΔ*wzm*	16M in-frame deletion mutant in *wzm*; R-LPS; polymyxin susceptible; internal O-PS	IdAB-CSIC
Rev1	B. melitensis bv1 reference vaccine strain; S-LPS	IdAB-CSIC
Rev1Δ*wzm*	Rev1 in-frame deletion mutant in *wzm*; R-LPS; polymyxin susceptible; internal O-PS	IdAB-CSIC
BoPA	B. ovis PA reference virulent strain; R-LPS	IdAB-CSIC
B. canis	B. canis RM6/66 virulent strain; R-LPS	IdAB-CSIC
2308	B. abortus bv1 reference virulent strain; S-LPS	IdAB-CSIC
S19	B. abortus bv1 reference vaccine strain; S-LPS	IdAB-CSIC
B. abortus bv1	B. abortus bv1 field isolates (*n* = 5) and 2308 from different origins (*n* = 3)	LCSA and IdAB-CSIC
B. abortus bv3	B. abortus bv3 field isolate; S-LPS	LCSA
RB51	B. abortus bv1 commercial vaccine strain; R-LPS	LCSA
B. suis bv1	B. suis bv1 1330 reference virulent strain; S-LPS	LCSA
B. suis bv2	B. suis bv2 field isolate; S-LPS	LCSA

a S-LPS, smooth lipopolysaccharide; R-LPS, rough lipopolysaccharide; IdAB-CSIC, Instituto de Agrobiotecnología (CSIC-Gobierno de Navarra); LCSA, Laboratorio Central de Sanidad Animal–National Reference Laboratory of Brucellosis (Santa Fe, Granada, Spain).

**(ii) The inhibition of Rev1Δ*wzm* in CM is due to the synergistic interaction between vancomycin and colistin.** After observing the strong inhibition of Rev1Δ*wzm* in CM, we selected this mutant to investigate the activity of the three antibiotics contained in this medium, i.e., vancomycin, colistin, and nitrofurantoin. The MIC and MBC values indicated that only vancomycin and colistin inhibited Rev1Δ*wzm* more than the parental strain; their activity against the mutant was synergistic, as opposed to additive for Rev1 (Table 2). We then sought to establish the optimal concentration of both antibiotics for Rev1Δ*wzm* growth by monitoring the number of CFU in CM plates containing different amounts of vancomycin and colistin versus BAB. As shown in Fig. 1B, 10 μg/mL of vancomycin combined with 4 μg/mL of colistin yielded similar growth to that in BAB, and these concentrations were therefore used in the reformulated Brucella selective medium (BSM) (Table 3).

**TABLE 2:** Susceptibility of Rev1Δ*wzm* and Rev1 to the antibiotics of CM

Strain	Antibiotic	Antimicrobial activity			
		Individual MIC/MBC_90_ (μg/mL)	Combined		
			MIC (μg/mL)	ΣFIC^*a*^	Effect^*b*^
Rev1Δ*wzm*	Vancomycin	320/640	40	0.37	Sinergy
	Colistin	46.9/93.8	11.7	0.37	Sinergy
	Nitrofurantoin	160/160	ND	ND	ND
Rev1	Vancomycin	640/640	320	1.00	Additive
	Colistin	93.8/187.5	46.9	1.00	Additive
	Nitrofurantoin	160/160	ND	ND	ND

a FIC, fractional inhibitory concentration index.

b ΣFIC < 0.5: synergy, ΣFIC = 0.5–1: additive. ND, not determined.

**TABLE 3:** Composition of FM, CM, and the novel *Brucella* selective medium (BSM) and *Bru*SIC selective solid media

Component	FM	CM	BSM	*Bru*SIC
Base^*a*^	BAB	BAB	BAB	BAB
Supplement^*b*^	5% NBCS	5% NBCS	5% NBCS	1 g/L activated charcoal
Antimicrobials	FM lyophilized supplement^*c*^	Drugs individually weighed and properly diluted^*d*^	New lyophilized supplement^*e*^	New lyophilized supplement
Antibiotics	V_20_	V_20_ in ultrapure water	V_10_	V_10_
	PxB_5_	Col_7.5_ in ultrapure water	Col_4_	Col_4_
	Nx_5_	Nitro_10_ in DMF	Nitro_10_	Nitro_10_
	Baci_25_			
Antifungals	Nysta_100_	Nysta_100_ in methanol	Nysta_100_	Nysta_100_
	Cyclo_100_	AmphoB_4_ in DMSO	AmphoB_4_	AmphoB_4_

a BAB, Blood Agar Base No. 2, at 40 g/L diluted in ultrapure water.

b 5% NBCS (newborn calf serum) at 50 mL/L or activated charcoal powder at 1 g/L.

c FM lyophilized antimicrobial supplement (commercial) were diluted in ultrapure water and contained V_20_ (20 mg/L of vancomycin), PxB_5_ (5,000 IU/L of polymyxin B), Nx_5_ (5 mg/L of nalidixic acid), Baci_25_ (25,000 IU/L of bacitracin), Cyclo_100_ (100 mg/L of cycloheximide), and Nysta_100_ (100,000 IU/L of nystatin).

d CM antimicrobials were individually weighed and diluted in the corresponding solvent to obtain V_20_ and Col_7.5_ (7.5 mg/L of colistin), both diluted in ultrapure water, Nitro_10_ (10 mg/L of nitrofurantoin diluted in *N*,*N*-dimethylformamide [DMF]), AmphoB_4_ (4 mg/L of amphotericin B diluted in dimethyl sulfoxide [DMSO]), and Nysta_100_ (100,000 IU/L of nystatin diluted in metanol).

e BSM and *Bru*SIC lyophilized antimicrobial supplement (prepared for this study) were diluted in ultrapure water and contained V_10_ (10 mg/L), Col_4_ (4 mg/L), Nitro_10_, Nysta_100_, and AmphoB_4_. For references to all products, see Materials and Methods.

### Performance of BSM for the isolation of *B. melitensis*.

**FIG 2:**
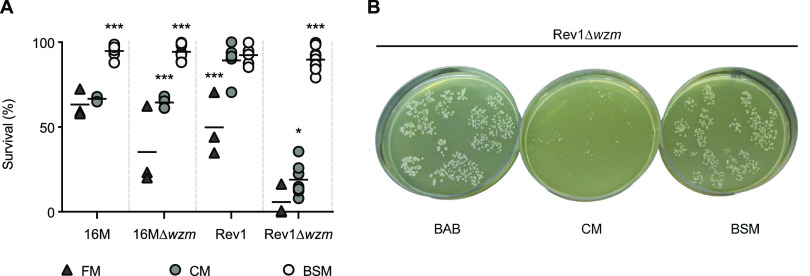
Growth of B. melitensis strains in FM, CM, and BSM. (A) Survival (% versus BAB) in each selective culture medium. Each symbol represents an independent experiment, and the horizontal bars indicate the mean value of the group. (B) Colonial size of Rev1Δ*wzm* in BAB, CM, and BSM plates after 5 days of incubation at 37°C. A Fisher LSD test was performed (***, *P* ≤ 0.001; *, *P* ≤ 0.05) versus other culture media for a given strain.

** (i) BSM improves the growth of 16M, 16MΔ*wzm*, and Rev1Δ*wzm* versus FM and CM.** To assess the performance of BSM, we determined the survival of different B. melitensis strains in this medium versus BAB, using FM and CM as reference media. As shown in Fig. 2A, BSM enabled the growth of all these B. melitensis strains at the levels of BAB, improving (*P* ≤ 0.001) the growth observed in the other two selective media, except for Rev1, which grew similarly in both BSM and CM. In detail, BSM yielded >90% recovery of 16M, 16MΔ*wzm*, and Rev1Δ*wzm* in contrast to the 67, 65, and 19% bacterial survival in CM, respectively. Moreover, BSM allowed faster bacterial growth than CM, as Rev1Δ*wzm* reached the same homogeneous colonial size (1 to 1.2 mm in diameter) in BSM as in BAB at 5 days of incubation compared to the 7 days of culture required to yield visible colonies in CM (Fig. 2B).

**(ii) BSM inhibits the growth of contaminant microorganisms as effectively as CM and FM.** The ability of BSM to inhibit contaminant microorganisms was determined with a collection of 15 bacterial species recruited from field veterinary samples (Table 4). Like CM, BSM totally inhibited the growth of 12 contaminant microorganisms at concentrations as high as 10^8^ to 10^10^ CFU/mL, even after 14 days of incubation. Long storage of both selective media plates for 8 weeks at 4°C caused a progressive loss of inhibition for Enterococcus faecalis, Staphylococcus aureus, Pseudomonas aeruginosa, and Proteus vulgaris.

**TABLE 4:** Inhibition of bacterial contaminants isolated from real veterinary samples in BSM and CM

Strain	Bacterial (CFU/mL)^*a*^
Listeria monocytogenes	1 × 10^10^
Streptococcus pyogenes	1 × 10^10^
Enterococcus faecalis	1 × 10^10^
Staphylococcus aureus	1 × 10^10^
Bacillus amyloliquefaciens	1 × 10^10^
Citrobacter freundii	1 × 10^9^
Escherichia coli	1 × 10^9^
Salmonella enterica	1 × 10^9^
Pseudomonas aeruginosa	10^8^ – 10^9^
Klebsiella pneumoniae	10^8^ – 10^9^
Enterobacter cloacae	10^8^ – 10^9^
Proteus vulgaris	1 × 10^8^
Proteus mirabilis	NI
Serratia liquefaciens	NI
Hafnia alvei	NI

a Bacterial concentration totally inhibited in both BSM and CM. NI, no inhibition at 1 × 10^3^ CFU/mL.

Also, a total of 1,789 field sheep samples (swabs and tissue homogenates in phosphate-buffered saline [PBS]) were cultured in BSM, CM, and/or FM using BAB as a control. The results indicated that BSM was as inhibitory as FM and CM for accompanying bacteria and fungi. In fact, we only detected contamination in 5.3% of BSM or CM and 4% of FM plates. The microorganisms occasionally found were identified by matrix-assisted laser desorption ionization–time of flight mass spectrometry (MALDI-TOF MS) as *Aerococcus* sp., Pseudomonas aeruginosa, Pseudomonas putida, Roseomonas mucosa, Corynebacterium freneyi, Corynebacterium xerosis, and Ochrobactrum intermedium.

**FIG 3:**
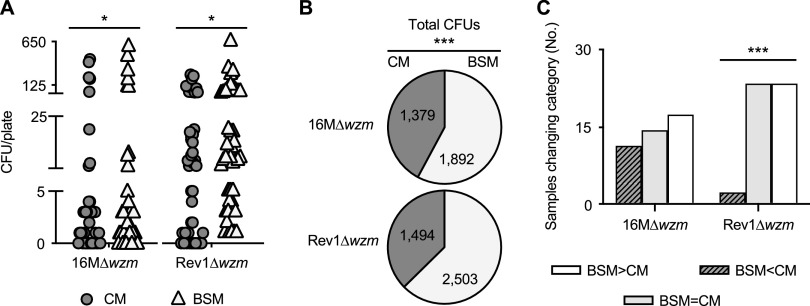
Performance of BSM versus CM for the isolation of *B. melitensis* 16MΔ*wzm* and Rev1Δ*wzm* mutants from sheep samples cultured in duplicate in both selective media. A total of 90 plates containing ≤650 CFU/plate of 16MΔ*wzm* (*n* = 42) or Rev1Δ*wzm* (*n* = 48) was selected for comparison purposes. (A) Individual counts per plate. (B) Total number of CFU. (C) Numbers of samples in which BSM increased (>), maintained (=), or reduced (<) the infection category. A paired *t* test (panel A) or a chi-squared test (panels B and C) was performed (***, *P* ≤ 0.001; *, *P* ≤ 0.05) between selective culture media for a given strain.

**(iii) BSM improves the primary isolation of 16MΔ*wzm* and Rev1Δ*wzm* in comparison to CM.** The performance of BSM versus CM was assessed by duplicate culture of the 1,789 field samples from sheep inoculated with 16MΔ*wzm* (391 samples), Rev1Δ*wzm* (1,111 samples), H38 (75 samples), or B. ovis PA (212 samples). The two culture media provided similar total numbers of CFU for B. ovis (4,716 in BSM versus 4,611 in CM) and H38 (1,644 in BSM versus 1,626 in CM), but BSM improved the detection of Rev1Δ*wzm* and 16MΔ*wzm* in comparison with CM (Table 5 and Fig. 3). As shown in Table 5, the mutants were found in a total of 100 samples, which were positive in at least one culture medium; 90 were positive in BSM and 74 in CM, 64 being positive in both media. A total of 90/100 samples provided from 1 to 650 CFU/plate, the results being readily quantifiable; 80 of the samples were positive in BSM and 64 in CM. Moreover, subtracting the number of samples positive in BSM from the number of samples positive in both media (Table 5) revealed that 26/90 (28.9%) samples (12 of Rev1Δ*wzm* and 14 of 16MΔ*wzm*) would have been assumed negative if using only CM. In fact, BSM allowed the growth of more CFU/plate than CM (*P* ≤ 0.05) when comparing paired individual samples (Fig. 3A), which was also demonstrated (*P* ≤ 0.001) by the total number of CFU in BSM versus CM (Fig. 3B); this difference indicates that BSM improved the productivity of CM by 78.2% for Rev1Δ*wzm* and 37.2% for 16MΔ*wzm*. Furthermore, 40/90 (44.4%) samples were classified in a higher category of infection in BSM than in CM, i.e., 23/48 (47.9%) of Rev1Δ*wzm* samples and 17/42 (40.5%) of 16MΔ*wzm* samples (Fig. 3C).

**TABLE 5:** Duplicate culturing in BSM and CM of field samples from sheep at different status or free of infection, after experimental inoculation with B. melitensis 16MΔ*wzm* or Rev1Δ*wzm* mutants

Strain	No. of samples								
	Cultured in both media	Positive^*a*^				Showing 1 to 650 CFU/plate^*b*^			
		BSM	CM	In both media	In at least one medium^*c*^	BSM	CM	In both media	In at least one medium^*c*^
Rev1Δ*wzm*	1,111	50	38	38	50	48	36	36	48
16MΔ*wzm*	391	40	36	26	50	32	28	18	42
Total	1,502	90	74	64	100	80	64	54	90

a Samples with at least 1 CFU/plate.

b Excluding positive samples yielding >650 CFU/plate.

c Total number of positive samples for each Brucella strain.

### Development of the *Bru*SIC medium to improve the isolation of different *Brucella* species.

**FIG 4:**
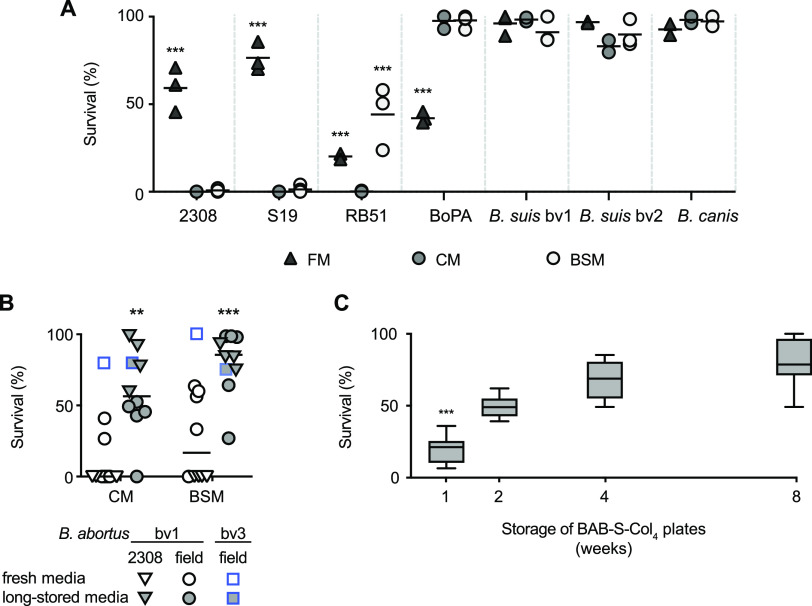
Survival of Brucella strains in selective culture media. (A) Survival of B. abortus, B. ovis PA, B. suis, and B. canis strains in FM, CM, and BSM versus the BAB control (or BAB-S for BoPA). Each symbol represents an independent experiment, and the horizontal bars are the mean values of the group. (B) Strains of B. abortus bv1 2308 from different origins (triangles; *n* = 4) or field isolates (circles; *n* = 5) and a bv3 field isolate (squares) grown in CM and BSM versus the BAB control, using either fresh media (within 1-week of preparation) or after long-term storage (8 weeks at 4°C). Each symbol represents one strain (by triplicate counting), and the horizontal bars are the mean value of the group (*n* = 10). (C) B. abortus bv1 2308 in BAB-S supplemented with 4 mg/L of colistin (BAB-S-Col_4_) versus the BAB control and used at different intervals from 1 to 8 weeks after storage at 4°C. Boxes represent the two central quartiles, the 10th to 90th percentile and the median of 6 to 9 replicates. A Fisher LSD test was performed (***, *P *≤ 0.001; **, *P* ≤ 0.01) versus other media or experimental conditions for a given strain.

**(i) BSM and CM inhibit the growth of *B. abortus* bv1 when freshly prepared, but not after 4 weeks of storage.** After improving B. melitensis isolation in BSM, we compared the suitability of this medium for the growth of B. abortus, B. ovis, *B suis*, and B. canis versus FM and CM (Fig. 4A). Except for B. abortus, the survival of all strains in BSM was similar to or better than in FM or CM. In fact, all three media improved the CFU recovery of B. suis and B. canis by >80% versus BAB, whereas the growth of B. ovis PA was higher in BSM and CM (98%) than in FM (41.9%). Strain B. ovis PA required at least 12 days of incubation to yield the total CFU, since only <3 CFU/plate of B. ovis PA were found at 1 week of incubation in FM.

Unexpectedly, B. abortus 2308, S19 and RB51 suffered a significant inhibition of growth in the three selective media versus BAB (Fig. 4A). The inhibition was more marked for RB51 than for strains 2308 and S19 in FM, complete for strains 2308 and S19 in BSM and CM, and RB51 grew better in BSM than in CM or FM. After performing repeated independent experiments to confirm these findings, we observed that inhibition of strain 2308 also occurred in a 10% CO_2_ incubation atmosphere (not shown), but not in BSM and CM plates stored at 4°C and used 8 weeks after preparation. To verify this result, we cultured eight additional B. abortus bv1 strains and one bv3 field strain (Table 1) in both BSM and CM, which were used either fresh (≤1 week after preparation) or long-stored (≥8 weeks after preparation) (Fig. 4B). Interestingly, while B. abortus bv3 exhibited >74% survival in both media regardless of plate storage time, all B. abortus bv1 strains were significantly more inhibited in fresh versus long-stored plates, more markedly in CM than in BSM (Fig. 4B).

To understand the reasons for this inhibition, we determined whether the BSM antibiotics were inactivated after 4 weeks of storage in the absence of water loss. For this, we compared the growth of strain 2308 in a liquid medium (tryptic soy broth [TSB]) supplemented with the BSM antibiotics, either freshly prepared or after 1 month of storage. The results showed the same loss of inhibition for strain 2308 as in BSM plates (data not shown). Likewise, the pH values (7 to 7.5) of BSM and CM plates did not change during the 4-week storage period studied. Subsequently, considering the high susceptibility to polymyxin B reported for some B. abortus strains (29), we studied the MIC of colistin for strain 2308, determining that it was 15.5 μg/mL. This value was considerably lower than that obtained for Rev1Δ*wzm* (46.9 μg/mL), which explains at least partially the observed inhibition. To shed more light on this finding, we examined the effect of plate storage on colistin activity, confirming that strain 2308 was inhibited in fresh BAB-S supplemented with 4 μg/mL of this antibiotic, but its survival increased weekly during storage (Fig. 4C). Nevertheless, we also observed that reduced doses or even total removal of colistin from BSM did not prevent strain 2308 inhibition in fresh plates (not shown), suggesting a possible interaction of the antibiotic and/or the strain with other antibiotics or components of BSM.

**FIG 5:**
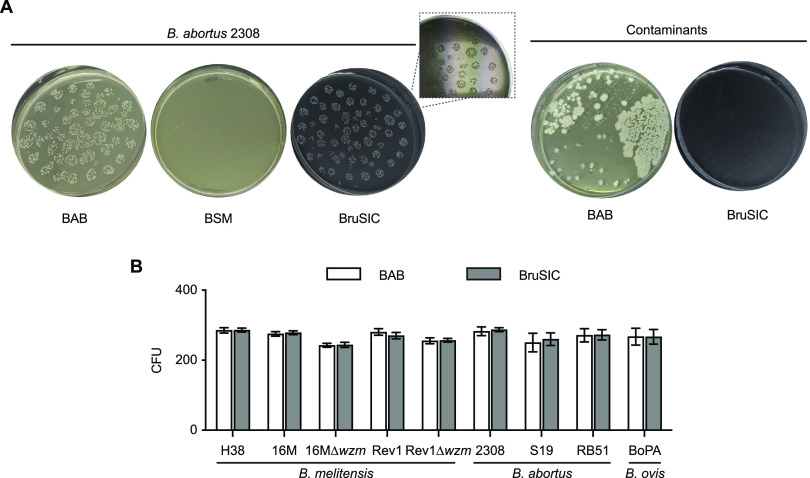
Productivity of *Bru*SIC for the isolation of different Brucella strains showing inhibition in other selective media. (A) Representative images of B. abortus bv1 strain 2308 grown in fresh BAB, BSM, and *Bru*SIC plates after 3 days of incubation (left) and successful inhibition of contaminant microorganisms from sheep milk samples in *Bru*SIC versus BAB (right). (B) Numbers of CFU determined by plating (100 μL, triplicate) in *Bru*SIC versus BAB (or BAB-S for BoPA) after 4 days of incubation. The results represent the mean CFU ± the SD (*n* = 3) counted in each culture medium.

**(ii) The replacement of BSM serum by activated charcoal in *Bru*SIC allows normal growth of *B. abortus* bv1 and promotes early development of *Brucella* colonies.** To understand whether the inhibition of B. abortus 2308 was due to charge instability, we studied the effect of replacing BSM serum with activated charcoal, as reported for other bacteria (30), since the negative surface of this component can interact with the cationic nature of polymyxins (31). In the presence of 1 to 4 g/L of activated charcoal, but not at lower concentrations, B. abortus 2308 grew at the levels of BAB. Accordingly, we selected 1 g/L for the new *Bru*SIC medium (Table 3), since this concentration also allowed a clear identification of the Brucella morphology through mildly translucent blackish plates. We systematically found a similar number of strain 2308 CFU in fresh *Bru*SIC and in BAB, with a successful inhibition of veterinary sample contaminant microorganisms in the former (Fig. 5A).

After solving the problem of B. abortus bv1 inhibition by the use of activated charcoal, we verified that *Bru*SIC performed as effectively as BAB for the isolation of brucellae previously inhibited in other selective media, i.e., B. abortus 2308, S19, and RB51; B. ovis PA; and B. melitensis H38, 16M, Rev1, 16MΔ*wzm*, and Rev1Δ*wzm* (Fig. 5B). Moreover, B. abortus, B. melitensis, and B. ovis colonies grew faster in *Bru*SIC than in BSM, providing a homogeneous size as in BAB at 2 to 4 days post-incubation. Interestingly, *Bru*SIC allowed the normal growth of B. ovis PA, proving that activated charcoal can be a functional substitute for serum in Brucella selective media.

Besides inhibiting the bacterial contaminants as effectively as BSM, *Bru*SIC successfully inhibited (*P* ≤ 0.001 versus BAB) the microorganisms from 61 ovine and 20 bovine field samples (tissues or milk) at an equivalent level to BSM (6/81 versus 11/81 contaminated plates; Table 6).

**TABLE 6:** Inhibition of contaminant microorganisms in BSM and *Bru*SIC selective media using cultures of highly contaminated real veterinary samples

Sample	No. of contaminated plates^*a*^		
	BAB	BSM	*Bru*SIC
Sheep tissues	19*	2	0
Sheep milk	42*	9	6
Cow milk	20*	0	0
Total	81*	11	6

a Samples with at least 1 CFU/plate. *, *P* ≤ 0.001 (Chi-square test) versus selective media. No significant differences were found between BSM and *Bru*SIC.

## DISCUSSION

Before starting safety experiments on Brucella infection in the natural host, the suitability of the selective culture media must be assessed to avoid biased results, such as false negatives or misclassified levels of infection. Accordingly, the initial aim of this study was to evaluate the growth of two B. melitensis R-LPS mutants in the recommended selective culture media FM and CM (1) due to their high susceptibility to polymyxins B and E (27, 28), which are present in FM and CM, respectively. As hypothesized, both FM and CM significantly inhibited the growth of both B. melitensis rough mutants, particularly Rev1Δ*wzm*. It was also found that the inhibition of Rev1Δ*wzm* in CM was related not only to colistin susceptibility but also to a synergistic effect between this polymyxin and vancomycin. As previously suggested, the synergy was probably due to vancomycin having increased access in the presence of colistin (32). The inhibitory effect of colistin against B. abortus bv1 disappeared progressively during storage of CM, attributable to their degradation in the presence of oxygen (31).

Besides the expected inhibition of 16M, Rev1, and B. ovis PA in FM (17, 18), we found that the virulent reference strain B. melitensis 16M was also inhibited in CM. Since this can influence the accuracy of results in natural hosts, CM should be modified, as has been done with FM for use in sheep (33). Overall, the different degrees of inhibition observed for B. melitensis, B. abortus, and B. ovis strains were in line with previous studies with FM (15, 17, 19, 34) or CM (35). All of these findings highlight the importance of medium composition when isolating a particular Brucella strain, even within the same species. The results prompted us to formulate BSM with half the doses of colistin and vancomycin found in CM, as shown in Table 3.

A validation experiment using a total of 1,789 sheep samples revealed that BSM selectivity matched that of CM, successfully inhibiting both fungal and bacterial contaminants, in agreement with previous reports (17, 19, 35–38). Although medium formulation is a key factor for the maximum isolation of Brucella from veterinary samples, other strategies—such as clean sampling and tracing the cultures on a daily basis (35), external disinfection of organ/lymph node samples, or the use of two combined culture media (1)—are also recommended practices. In addition, BSM improved the primary isolation of attenuated B. melitensis mutants, providing a higher number of CFU compared to CM in experimental conditions and allowing colony detection in sheep samples that otherwise would have been recorded as free of infection or misclassified in a lower category.

Since significant inhibition of B. abortus bv1 in CM has not been reported before, we investigated its cause. On the one hand, we confirmed the previously described high susceptibility of B. abortus to colistin (29). Indeed, various studies focusing on polymyxin stability have reported physicochemical modifications and degradation over time (39–43). Also, these polycations are known to adhere to organic and inorganic materials, such as polystyrene plates (44, 45), as well as to exhibit slow diffusion through agar (43, 46). Furthermore, unpredictable compensatory mechanisms may be activated, involving interactions between medium components and bacterial dynamics (32). For example, the serum may exert a paradoxical effect on colistin activity, either synergistic or antagonistic, depending on the bacterial strain (47). However, susceptibility to colistin was not the only cause of inhibition, which was still observed after the reduction in concentration or even total removal. Interestingly, we found that B. abortus inhibition was directly related to the interval between the preparation and the use of CM and BSM plates. This finding may explain, at least partially, why this issue has not been reported previously. The consequences of storage should therefore be taken into consideration in procedures for primary isolation of Brucella, not only for the determination of field strains but also the evaluation of vaccine candidates.

To solve the problem of B. abortus bv1 inhibition, we designed the new *Bru*SIC medium containing activated charcoal, whose key role as a radical and peroxide scavenger has been proposed in *Legionella* media, such as BCYE and GVPC (31, 48). In addition to a higher recovery of CFU, the activated charcoal promoted a faster growth of Brucella colonies in *Bru*SIC, avoiding false-negative results arising from insufficient incubation, as described for B. ovis or B. suis cultures in FM (17, 19). Another previously proposed strategy to promote Brucella growth in selective culture media is the addition of enrichment components such as erythritol (34, 49). Interestingly, we observed that the substitution of calf serum by activated charcoal in *Bru*SIC allowed the successful isolation of more demanding Brucella strains, such as B. ovis PA. It also resulted in a less costly medium, requiring less manipulation, and did not impair the straightforward identification of Brucella colonies. In addition, the use of activated charcoal (of plant origin) instead of calf serum (animal source) represents a sustainable alternative, promoting animal welfare and reducing diagnostic costs, an essential factor in brucellosis control.

This is not the first attempt to modify CM formulation to improve its productivity (38). However, by reducing the antibiotic concentration and incorporating activated charcoal in *Bru*SIC, our strategy is preferable to the inclusion of alternative and/or additional antibiotics in selective media, as proposed in other studies (34, 38). The presence of multiple antimicrobials in selective culture media is a source of undetermined interactions among the components, favoring the emergence of resistant pathogens (50). Moreover, at the selected concentration of activated charcoal (1 g/L), *Bru*SIC was as inhibitory as CM and BSM for microorganisms usually present in veterinary samples. It was also sufficiently translucent for bacteria to be visualized through the bottom of the plate and with the help of backlighting. Moreover, the bright Brucella colonies stood out clearly from the black background, allowing the category of infection of the sample to be assigned at a glance. Higher concentrations of activated charcoal were considered unsuitable, since they allowed the growth of contaminant microorganisms and impaired the translucence for Brucella colony identification. These facts, together with the faster growth of all brucellae colonies in *Bru*SIC than in FM, CM, and BSM, indicate that *Bru*SIC can be highly recommended as an alternative medium (34, 37, 49). Activated charcoal has been occasionally used in bacterial culture due to its detoxifying properties (30, 51, 52), but to our knowledge it has never been applied for the identification of Brucella. The presence of activated charcoal in *Bru*SIC had a positive action that could explain not only the stabilization of charges affecting antibiotic activity but also a detoxifying effect, allowing the growth of B. ovis PA in the absence of calf serum. Indeed, it has been suggested that activated charcoal can interact with reactive oxygen species secondarily generated by antibiotics or during medium preparation in a normal atmosphere, as well as with the positive charges of polymyxins and/or vancomycin, especially in the presence of oxygen (31). These free radicals could also partially degrade vancomycin, promoting the growth of the mildly resistant Gram-positive E. faecalis (31). Hence, the addition of activated charcoal would explain the stabilization of both colistin and vancomycin, which we observed exerted a synergistic effect against the R-LPS mutant.

Altogether, our results strongly support the use of *Bru*SIC as a stable selective medium able to promote a faster growth of Brucella, including serum-dependent species, for primary isolation from veterinary field samples. In order to validate *Bru*SIC, additional studies are being conducted in animal samples (organ tissues and milk) previously contaminated with brucellae in a blind manner, as well as in real field samples obtained from different animal species infected with Brucella.

## MATERIALS AND METHODS

### Bacteria, medium preparation, and culture conditions.

The Brucella strains and contaminant microorganisms used here are listed in Tables 1 and 4, respectively. All bacteria were stored at −20°C in 10% skimmed milk supplemented with 3% lactose (both PanReac AppliChem, Barcelona, Spain). Routine culturing was performed in Blood Agar Base No. 2 (BAB; catalog no. CM0271; Oxoid, Ltd., Hampshire, UK) for Brucella and in TSA prepared with trypticase soy broth (TSB; Condalab, Madrid, Spain) supplemented with 1.5% bacteriological agar (Pronadisa, Madrid, Spain) for contaminant bacteria. Liquid cultures were performed in TSB at 150 rpm and 37°C. When required, media were supplemented with 5% newborn calf serum (NBCS; Gibco, Auckland, New Zealand; BAB-S), activated charcoal powder (Omya Clariana, Spain), and/or the antimicrobial agents (all from Sigma-Aldrich, Madrid, Spain) vancomycin (catalog no. V2002), colistin (catalog no. C1511), nitrofurantoin (catalog no. N7878), nystatin (catalog no. N3503), and amphotericin B (catalog no. A4888).

FM and CM were prepared in BAB-S, as described previously (17). FM was prepared with a commercially available lyophilized antimicrobial supplement (Oxoid, Ltd., catalog no. SR0083A, or Condalab, Madrid, Spain, catalog no. 6017) according to the manufacturer’s instructions. For CM preparation, each antimicrobial agent was individually weighed and diluted at the desired concentrations in the appropriate solvent (Table 3), i.e., vancomycin and colistin in ultrapure water, nystatin in methanol (VWR Chemicals), nitrofurantoin in *N*,*N*-dimethylformamide (DMF; Sigma-Aldrich), and amphotericin B in dimethyl sulfoxide (DMSO; VWR Chemicals). BSM and *Bru*SIC were prepared either with the antimicrobials prepared as for CM or, when available, with a lyophilized antimicrobial supplement specifically prepared for this work, and reconstituted in ultrapure water (10 mL/vial, 1 vial/500 mL of medium).

Bacterial suspensions were prepared in sterile phosphate-buffered saline solution (PBS [pH 7.2]; components from VWR Chemicals, Leuven, Belgium) by spectrophotometry adjustment (SmartSpec Plus Spectrophotometer, Bio-Rad, Hercules, CA) to 10^9^ CFU/mL, as described elsewhere (53). The exact number of CFU/mL was determined retrospectively by six serial 10-fold dilutions in PBS (from 10^9^ to 10^3^ CFU/mL), plating (100 μL, in microdrops, triplicate) of the last two dilutions (containing ≈10^4^ and 10^3^ CFU/mL, respectively), and incubation of plates at 37°C in normal air or under 10% CO_2_ atmosphere, for 7 days in BAB and BAB-S or up to 14 days in selective culture media. Plates containing ≈100 CFU/100 μL (lower and upper limits of 30 to 300 CFU/100 μL) were used to calculate the mean CFU/mL ± the standard deviations (SD; *n* = 3) or the numbers of CFU/100 μL. When indicated, the percentage of bacterial survival in the corresponding culture medium was determined versus BAB (or BAB-S for B. ovis PA) by seeding (as before) each of the seven 10-fold serial diluted bacterial suspensions, and incubation of plates at 37°C in a normal atmosphere (or 10% CO_2_, for B. ovis PA). Each experiment was performed at least twice independently.

### Susceptibility of *B. melitensis* strains to FM and CM and to antibiotics.

The inhibition of B. melitensis 16M, 16MΔ*wzm*, Rev1, and Rev1Δ*wzm* strains was assessed by culturing (37°C, 7 days) ≈300 CFU/100 μL in triplicate in FM, CM, and the BAB control. The mean number of CFU ± the SD (*n* = 3) for each bacterial strain in each culture medium was calculated as detailed above.

To determine the inhibition induced by the antibiotics in CM, individually and combined, we used the standard microdilution broth method, as described previously (27, 54), and chequerboard titration (55). Briefly, 2-fold serial dilutions of vancomycin (0.625 to 640 μg/mL), colistin (0.25 to 750 μg/mL), and nitrofurantoin (0.625 to 320 μg/mL) were prepared in cation-adjusted Müller-Hinton medium (M-H; BD, Heidelberg, Germany), immediately dispensed (100 μL/well) in 96-well polystyrene plates (Sarstedt, Nümbrecht, Germany) and mixed with an equal volume of bacterial suspension adjusted to optical density at 750 nm of 0.109 (≈1.5 × 10^9^ CFU/mL) and diluted 1:100 in M-H. Each concentration was analyzed in duplicate, and plain M-H wells with or without bacteria were used as controls. The minimum inhibitory and bactericidal concentrations (MIC and MBC_90_) were determined after incubation (at 37°C for 48 to 72 h). The fractional inhibitory concentration index (ΣFIC) of the combined antibiotics was calculated as follows: ΣFIC = (MIC of antimicrobial A combined/alone) + (MIC of antimicrobial B combined/alone), and was interpreted as follows: synergy (<0.5), additive effect (0.5 to 1.0), indifferent action (1.1 to 2.0), or antagonism (>2.0) (55).

### Definition of BSM and its performance with field sheep samples for the isolation of Rev1Δ*wzm* or 16MΔ*wzm*.

To formulate the BSM, ≈30 CFU/100 μL of Rev1Δ*wzm* were cultured in triplicate in CM prepared with different concentrations and combinations of vancomycin (20, 15 or 10 μg/mL) and colistin (8, 6 or 4 μg/mL). The mean CFU ± the SD (*n* = 3) obtained in each culture medium and in BAB were calculated as described above.

Once the optimal concentrations of vancomycin and colistin for BSM were selected (Table 3), we assessed the bacterial survival of B. melitensis 16M, Rev1, 16MΔ*wzm*, and Rev1Δ*wzm* in BSM, FM, and CM versus the BAB control. For this, we seeded (100 μL, in triplicate) each of the seven 10-fold serial dilutions of each strain suspension in each medium, and we calculated the mean CFU/mL ± the SD (*n* = 3) in each medium and the percent bacterial survival versus BAB in at least three independent experiments, as described above.

The performance of BSM for the primary isolation of Rev1Δ*wzm* and 16MΔ*wzm* mutants was determined by duplicate culturing (at 37°C for 14 days) of 1,502 samples from 141 experimentally infected sheep; CM and BAB were used as controls. The cultured samples were swabs impregnated in vaginal fluid, semen, milk, amniotic fluid, cotyledons, or fetuses, as well as in liver, spleen, seminal vesicle, epididymis, uterus, mammary gland, and lymph node tissues, all of which were obtained, processed, and homogenated in PBS as previously described (27). The identity of the presumptive 16MΔ*wzm* and Rev1Δ*wzm* colonies was confirmed by subculturing them in BAB, and further analysis was carried out to differentiate Rev1 from 16M and to detect the deletion in *wzm* by PCR-RFLP and PCR-WZM, as previously described (27, 56). The number of CFU/plate was used to determine the percentage of increasing productivity as follows: [(CFU in medium A − CFU in medium B)/CFU in medium B] × 100. Also, plates were classified according to several infection categories: 0 (no CFU found), 1 (1 to 5 CFU), 2 (6 to 25 CFU), 3 (26 to 125 CFU), 4 (126 to 650 CFU), or 5 (>650 CFU), and the number of BSM plates classified in higher, equal, or lower infection categories than CM was also recorded. Also, the contaminant microorganisms found in selective media were identified by MALDI-TOF MS at the Services of the Universidad Complutense de Madrid (UCM) as described elsewhere (57) and used for selectivity studies.

The selectivity of BSM was determined by recording the number of contaminated plates after culturing a total of 1,789 veterinary sheep samples in duplicate in BSM and CM; 75 of them were also checked in FM.

### Growth of other brucellae in BSM, FM, and CM.

The percent bacterial survival of B. abortus bv1 2308, S19, and RB51, B. suis bv1 1330 and bv2, B. ovis PA, and B. canis RM6/66 (Table 1) was assessed in plates of BSM, FM, and CM versus BAB (or BAB-S for B. ovis PA). After observing the inhibition of B. abortus bv1 strains in BSM and CM, we performed a similar study with eight additional B. abortus bv1 and one bv3 field strains in both selective culture media, either freshly prepared (within 1 week) or after 8 weeks of storage at 4°C (long-stored media). We also determined the effect of incubation in a 10% CO_2_ atmosphere by determining the percent survival of B. abortus 2308 in both BSM and CM versus BAB.

To elucidate the properties of BSM over time, we evaluated the medium pH (pH indicator strips; Merck, Darmstadt, Germany) after 1, 2, 3, and 4 weeks of plate storage at 4°C. Also, changes in medium properties due to dehydration were analyzed by determining the MIC of the antimicrobials in BSM for B. abortus 2308 by the microdilution broth method described above, with some modifications. Briefly, the antimicrobials were added to TSB or TSB-S, which were used freshly prepared or after 4 weeks of storage, at the same concentration as in the solid selective medium and at 2-fold dilutions; Rev1 was used as a control of noninhibition.

Thereafter, we evaluated the susceptibility of B. abortus bv1 strain 2308 to 4 μg/mL colistin during the storage time by determining the percent bacterial survival in BAB-S plates with colistin at 4 μg/mL (BAB-S-Col_4_), either freshly prepared (within 1 week) or after 2, 4, and 8 weeks of storage at 4°C. In addition, strain 2308 dilutions were cultured in fresh BSM containing 3, 2, 1, 0.5, or 0 μg/mL of colistin.

In all these experiments, the percent bacterial survival was determined as detailed above by culturing seven bacterial suspensions of the corresponding strain prepared by 10-fold serial dilutions in each culture medium versus BAB (or BAB-S for B. ovis PA) in at least two independent experiments.

### Definition and performance of *Bru*SIC.

To solve the problem of B. abortus bv1 inhibition, we tested activated charcoal powder diluted at different concentrations (from 0.1 to 4 g/L) in BAB supplemented with the mix of antimicrobials selected for BSM and in the absence of NBCS. The percentage of strain 2308 survival was determined by seeding seven 10-fold serial dilutions in each culture medium versus BAB, as detailed above.

Finally, *Bru*SIC was formulated with 1 g/L of activated charcoal. To assess its productivity, the growth of B. melitensis H38, 16M, Rev1, 16MΔ*wzm*, and Rev1Δ*wzm* strains, B. abortus 2308, S19, and RB51 strains, and B. ovis PA strains was assessed by triplicate plating of ≈300 CFU/100 μL in *Bru*SIC and BAB (or BAB-S for B. ovis PA). The mean number of CFU ± the SD (*n* = 3) for each strain grown in each culture medium was determined as described above.

The ability of *Bru*SIC and BSM to inhibit contaminant microorganisms was analyzed with a laboratory collection of 15 bacteria isolated from veterinary samples (Table 4), as well as with highly contaminated real field samples (swabs and tissue homogenates). For contaminant bacteria, seven 10-fold dilutions of each overnight culture at 37°C in TSB (≈10^10^ CFU/mL) were seeded (100 μL/plate, triplicate; 37°C, 14 days) in CM, BSM, and *Bru*SIC versus TSA control plates, used either freshly prepared or after storage at 4°C for 1, 2, 3, 4, 8, or 12 weeks; the highest completely inhibited bacterial concentration was recorded. For real veterinary samples, a total of 81 samples from Brucella-free sheep (milk *n* = 42 and tissues *n* = 19) or cows (milk *n* = 20) were cultured in *Bru*SIC, BSM, and BAB to determine the inhibition of contaminant microorganisms in each medium, recording the number of contaminated plates in each culture medium.

### Statistical analysis and graphical representation.

Statistical analysis and graphical representations were performed with Prism 8.4.0 software (GraphPad, Inc., San Diego, CA). Significance was determined by using a chi-square test, a paired one-tailed Student *t* test, or one- or two-way analysis of variance, followed by a Fisher least significant difference test with 95% confidence intervals, according to data classification. The final figures were assembled using the GNU Image Manipulation Program v. 2.10 (GIMP, open source, www.gimp.org) and Illustrator 2020 (Adobe, San José, CA).
